# Using multi-year national survey cohorts for period estimates: an application of weighted discrete Poisson regression for assessing annual national mortality in US adults with and without diabetes, 2000–2006

**DOI:** 10.1186/s12963-016-0117-x

**Published:** 2016-12-15

**Authors:** Yiling J. Cheng, Edward W. Gregg, Deborah B. Rolka, Theodore J. Thompson

**Affiliations:** Division of Diabetes Translation, Centers for Disease Control and Prevention, 4770 Buford Hwy, NE, MS F-73, Atlanta, GA 30341 USA

**Keywords:** Complex survey, Surveillance, Mortality, Survival analysis, Discrete survival time, Poisson regression

## Abstract

**Background:**

Monitoring national mortality among persons with a disease is important to guide and evaluate progress in disease control and prevention. However, a method to estimate nationally representative annual mortality among persons with and without diabetes in the United States does not currently exist. The aim of this study is to demonstrate use of weighted discrete Poisson regression on national survey mortality follow-up data to estimate annual mortality rates among adults with diabetes.

**Methods:**

To estimate mortality among US adults with diabetes, we applied a weighted discrete time-to-event Poisson regression approach with post-stratification adjustment to national survey data. Adult participants aged 18 or older with and without diabetes in the National Health Interview Survey 1997–2004 were followed up through 2006 for mortality status. We estimated mortality among all US adults, and by self-reported diabetes status at baseline. The time-varying covariates used were age and calendar year. Mortality among all US adults was validated using direct estimates from the National Vital Statistics System (NVSS).

**Results:**

Using our approach, annual all-cause mortality among all US adults ranged from 8.8 deaths per 1,000 person-years (95% confidence interval [CI]: 8.0, 9.6) in year 2000 to 7.9 (95% CI: 7.6, 8.3) in year 2006. By comparison, the NVSS estimates ranged from 8.6 to 7.9 (correlation = 0.94). All-cause mortality among persons with diabetes decreased from 35.7 (95% CI: 28.4, 42.9) in 2000 to 31.8 (95% CI: 28.5, 35.1) in 2006. After adjusting for age, sex, and race/ethnicity, persons with diabetes had 2.1 (95% CI: 2.01, 2.26) times the risk of death of those without diabetes.

**Conclusion:**

Period-specific national mortality can be estimated for people with and without a chronic condition using national surveys with mortality follow-up and a discrete time-to-event Poisson regression approach with post-stratification adjustment.

## Background

National surveillance of incidence, prevalence, and mortality is key to guiding and evaluating progress in chronic disease control and prevention. The prevalence of a chronic disease like diabetes can be affected by increasing incidence among persons without the disease as well as decreasing mortality among persons with the disease. Good estimates of mortality among persons with a chronic disease improve understanding of secular changes in prevalence, incidence, and mortality and their relationships. Since diabetes is often not recorded on death certificates as a direct, underlying, or contributing cause of death, the impact of diabetes on deaths in the United States population could be underestimated [1, 2]. The linkage of nationally representative surveys that include baseline disease status with mortality follow-up provides the opportunity to examine all-cause and cause-specific mortality among persons with diabetes or other chronic conditions.

From the policymaking and resource allocation perspectives, a cross-sectional estimate of mortality by calendar period (e.g., year) is highly desirable. Analyses of mortality follow-up data typically use survival approaches to examine the association between risk factors and death. In these analyses, the data are analyzed as a cohort covering the entire follow-up period, and the hazard of death is estimated for the cohort. However, this approach does not permit estimation of the hazard of death across time periods, nor does it provide valid annual or other calendar period estimates.

By following the conceptual framework of age-period-cohort analysis (APC) as represented by the Lexis diagram, multi-year cohort data can be decomposed into discrete time-to-event data and aggregated by calendar period [3, 4]. Calendar period all-cause mortality rates can be calculated by simply using the total number of deaths divided by the total person-years in each calendar period. Poisson regression, a generalized linear model, is appropriate for modeling unadjusted and adjusted mortality rates of multiple periods [4]. Discrete Poisson regression yields identical estimates to the piecewise exponential model, which is another alternative to the Cox proportional hazards model [5]. Nevertheless, most discrete time-to-event studies use aggregated group data and categorized independent variables [6]; we are not aware of previous publications using discrete Poisson regression applied to multi-year mortality follow-up data from national sample surveys.

In this study, to increase the awareness of estimating cross-sectional period mortality using multi-year national survey mortality follow-up data, we describe the construction of discrete survival time data in detail and demonstrate our approach from data preparation to data analysis. With diabetes as an example, we use population-weighted Poisson regression to model discrete survival time and estimate annual all-cause mortality by diabetes status. The US National Health Interview Survey (NHIS) 1997–2004 with mortality follow-up up to 2006 was used to illustrate this approach; US mortality estimates from the National Vital Statistics System (NVSS) were compared for validation purposes.

## Methods

### Continuous time-to-event data

**Fig. 1:**
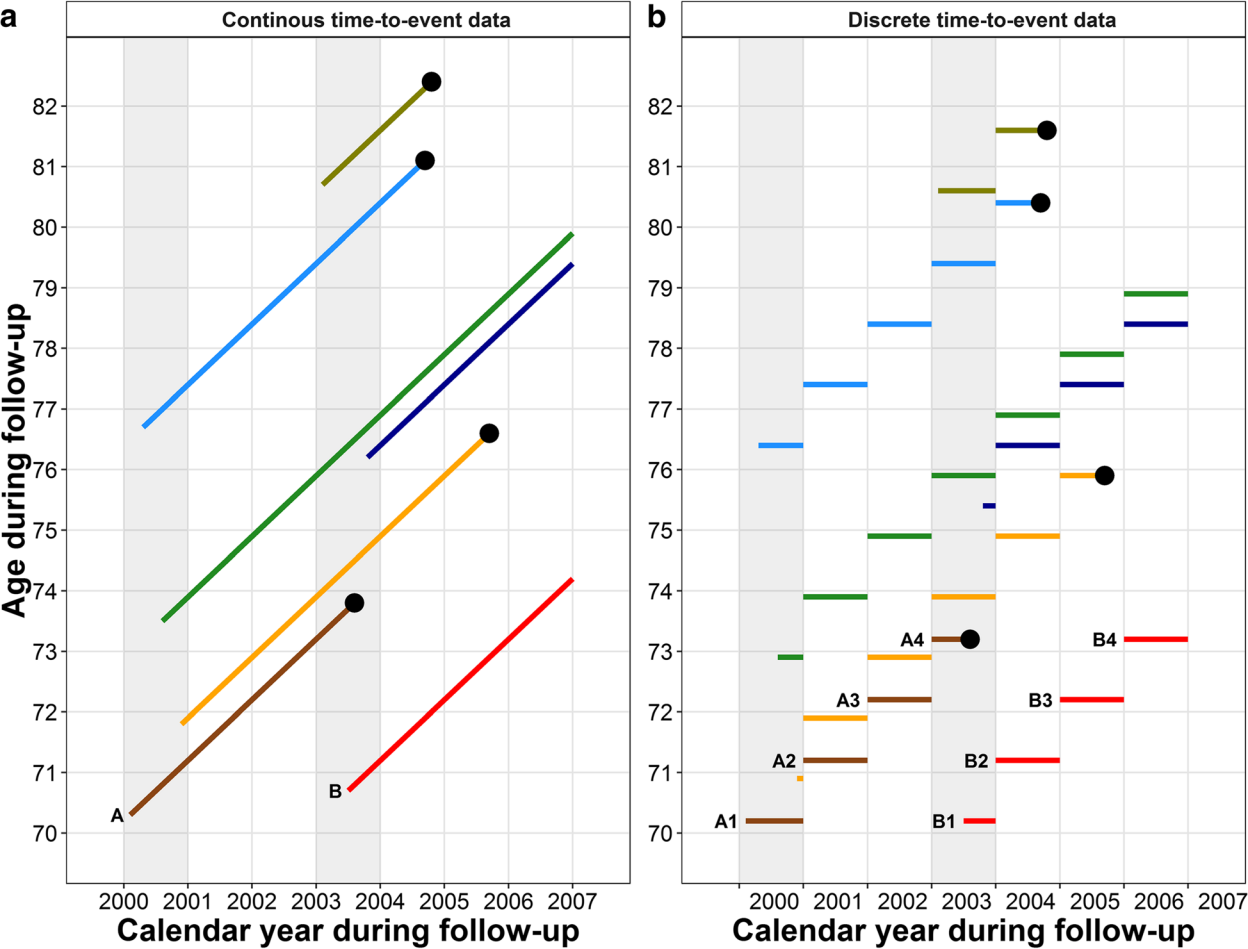
Follow-up of seven participants from 2000–2006 in continuous (1**a**) and discrete (1**b**) time-to-event format. The dot at the right end of lines represent death. Four participants were interviewed in 2000 and three participants were interviewed in 2003. All seven participants were followed up to the event or end of year 2006. For part 1a, both calendar year and age during the follow-up are shown as continuous variables. For part 1b, the time-to-event was split yearly; the y-axis shows the discretized age during the follow-up calendar year using the age at the first day of the year. The discrete age increased yearly with the follow-up

Survival analysis studies the occurrence and timing of events. Individual time-to-event data includes three components: the time of study entry (t_0_), the time of study exit (t_1_), and the event (i.e., death (D) or censoring (C)). In this study, the total follow-up time is the difference between the end of follow-up (t_1_, date of death or date of censor, whichever came first) and the date of the NHIS baseline interview (t_0_). We used a modified Lexis diagram to demonstrate the structure of continuous time-to-event survival data (Fig. 1 – 1a) [4]. Each participant (i)’s follow-up experience, represented by the diagonal line segment [from (year_t_0i_, age_t_0i_) to (year_t_1i_, age_t_1i_)], is shown on the plot of age versus calendar year. For clinical trials with a short follow-up time and matched age, follow-up time is usually used as the time scale in the survival analysis. For observational epidemiological studies with much longer follow-up time and a diverse age distribution of the observed sample, it has become popular to use age during follow-up as the time scale [7–9]. Typical continuous time-to-event data have a single record for each participant (Table 1 - Part I). For example, Person A entered the cohort at 02/06/2000 and had a total follow-up time of 3.5 years; person B entered the cohort at 07/02/2003 and had a total follow-up time of 3.5 years as well.

**Table 1:** Demonstration of continuous and discrete time-to-event survival formats

Part I: Continuous time-to-event format									
Person (i)	Date of birth	Date of interview (t0i)	Data of death or censored (t1i)	Discretized time period	Follow-up (years) (Ti)	Age at interview (years) (age at t0i)	Calendar year at interview (year at t0i)	Event status (1 = died) (0 = lived) (Di)	Sex (1 = Male) (0 = Female)
A	10/01/1929	02/06/2000	08/08/2003	---	3.5	70.3	2000	1	1
B	11/07/1932	07/02/2003	12/31/2006	---	3.5	70.7	2003	0	0
Part II: Discrete time-to-event format									
Person (i) & time period (j)	Date of birth	Date of interview (t0ij)	Data of death or censored (t1ij)	Discretized time period (ij)	Year of follow-up (Tij)	Age at the beginning of year of follow-up	Calendar year during follow-up (year at ij)	Event status (1 = died) (Dij)	Sex (1 = Male) (0 = Female)
A1	10/01/1929	02/06/2000	12/31/2000	1	0.9	70.2	2000	0	1
A2		01/01/2001	12/31/2001	2	1.0	71.2	2001	0	1
A3		01/01/2002	12/31/2002	3	1.0	72.2	2002	0	1
A4		01/02/2003	08/08/2003	4	0.6	73.2	2003	1	1
B1	11/07/1932	07/02/2003	12/31/2003	1	0.5	70.1	2003	0	0
B2		01/01/2004	12/31/2004	2	1.0	71.1	2004	0	0
B3		01/01/2005	12/31/2005	3	1.0	72.1	2005	0	0
B4		01/01/2006	12/31/2006	4	1.0	73.1	2006	0	0

### Discrete time-to-event survival data

To calculate a period-specific mortality rate, we divided the continuous survival times into discrete calendar years. Since interviews did not all take place on the first day of the survey year, to make sure the survival time was allocated correctly we added an individual-specific partial time period (t_ext_i_), calculated as the difference between the interview date and the first day of the year, then we divided the extended survival time [(t_1_−t_0_) + t_ext_i_] into years. That is, each person’s total continuous survival time was discretized into multiple records, one for each calendar year. An individual’s survival time for a given calendar year was between 0 and 1 year, and the survival time in the first year was [1–t_ext_i_]. In the analysis, age and calendar year were treated as time-varying (i.e., time-dependent) covariates. The age during each discrete period was assigned as the age on the first day of that calendar year.

With each participant contributing multiple discrete person-years during the follow-up, the sum of a person’s discretized annual person-years is equal to the total continuous survival time of that person (Fig. 1 – 1b). For example in Table 1 – Part II, person A was interviewed on 02/06/2000 at age 70.3 years and contributed 3.5 person-years of follow-up. That is, person A contributed 0.9 person-years in 2000 with age of 70.2 years at the beginning of year 2000, contributed 1 person-year in each of years 2001 and 2002 with age of 71.2, and 72.2 years at the beginning of those years, respectively, and in 2003, person A contributed 0.6 person-years before dying on 08/08/2003 at age 73.2 years at the beginning of the year. In this way, the continuous time-to-event survival records of participants have been decomposed into discrete survival time with time-varying age.

### US national health interview survey and mortality follow-up

We used the NHIS mortality follow-up data to demonstrate our approach. The NHIS, conducted by the Centers for Disease Control and Prevention’s National Center for Health Statistics (NCHS), is an annual ongoing nationally representative cross-sectional household interview survey of US non-institutionalized civilians of all ages [10]. The sampling plan covers the 50 states and the District of Columbia, and follows a multistage area probability design that permits the representative sampling of households and non-institutional group quarters. The annual response rate of NHIS is approximately 80% of the eligible households in the sample [10]. All information about sex, race/ethnicity (non-Hispanic white, non-Hispanic black, Hispanic, and others), and diabetes status was self-reported. Participants were classified as having diabetes if they answered “yes” to the question “Other than during pregnancy, have you EVER been told by a doctor or other health professional that you have diabetes or sugar diabetes?”

For our analysis, we selected the NHIS 1997 to 2004 surveys as the baseline, with mortality follow-up up through 2006; the NHIS 2005 to 2006 surveys were used to obtain the demographic distribution for the post-stratification reweighting of those two years. We included 307,280 adults aged 18 to 84 years from the NHIS 1997 to 2004 (range: 30,141 to 35,437 per year) and followed them through 2006. We excluded 15,882 (range: 1,723 to 2,642 per year) respondents because of insufficient identifying data to create a death status record, which yielded a final mortality follow-up sample of 242,397 (range: 29,076 to 29,193 per year) adults. The mortality follow-up sampling weights provided by NCHS accounted for excluded respondents.

Diabetic death was defined as a death with an associated International Classification of Diseases, 10th Revision (ICD-10) code of E10-E14. All-cause with diabetes death was defined as a person with diabetes who died of any cause. The total weighted person-time was used as the denominator for mortality calculation. We also estimated mortality by self-reported diagnosed diabetes at baseline. To validate our findings empirically, we compared the all-cause and diabetic mortality rates from NHIS with mortality rates from the NVSS, the fundamental source of US cause-of-death information. Mortality rates from the NVSS were directly calculated as number of death (all-cause or diabetic death coded as E10 to E14) divided by total population using structured query language from CDC WONDER by following the step-by-step instruction on the WONDER website (http://wonder.cdc.gov/mortSQL.html).

To reduce potential selection bias due to respondents being healthier than non-respondents, we excluded each individual’s first two years of follow-up. The final analytical discrete time-to-event data set included adults aged 20 years or older during the years 2000 to 2006.

### Poisson regression

Poisson regression was used to analyze and estimate the mortality rate [11]. The mortality (hazard) rate can be estimated using the following equation when follow-up times (*pt*) vary across individuals:

$$ \begin{array}{l} \log (d)= \log (pt)+{\beta}_0+{\displaystyle \sum {\beta}_i{X}_i,\kern0.5em }\\ {}\mathrm{then},\kern0.5em  mortality\kern0.5em  rate=d/ pt= \exp \left({\beta}_0+{{\displaystyle \sum {\beta}_iX}}_i\right)\end{array} $$

Here, the natural logarithm of the expected value of the event, log(*d*), with an offset of natural logarithm of follow-up time, log(*pt*), is a linear combination of independent covariates, *X*_*i*_, with regression parameters *β*_*i*_,.

Poisson regression provided the estimate of mortality for each calendar year/period. We used the robust error variances estimation approach to minimize over-dispersion [12] and the polynomial function of calendar time to smooth year-to-year variation in mortality rates [6, 13]. To smooth the variation in mortality due to low mortality rates in some age subgroups, the age at the beginning of a calendar year was defined as a continuous variable with polynomial terms (quadratic polynomial). The mortality rates in our study were estimated by the predictive margins of the regression coefficients from the Poisson model.

### Adjusted sampling weights for the discrete time-to-event data

The age of sampled participants in each survey cohort increased with the year of follow-up and those multi-year survey cohorts also overlapped time periods. Without accounting for the demographic discrepancy between the participants from different cohorts and the US population at each specific year, the demographic distribution of a discrete period after the baseline year would not represent the demographic distribution of the US population at that specific year or time-period, and the total crude mortality of the US population would be biased toward the older population. In order to correct for these issues, we adjusted the sample weights using a post-stratification procedure in which sampled units were divided into subgroups based on age, sex, and race/ethnicity; we used the nationally representative weighted size of each subgroup of NHIS 2000 to 2006 at interview to estimate the US population size. The analysis weights for the discrete time-to-event data were reweighted proportionally. The adjusted analysis weights thus sum to the US population size within each subgroup. The sum of the analysis weights equaled the total non-institutionalized US population for each calendar year.

### Analysis

We used Stata 13.1 (StataCorp LP, College Station, Texas) to account for the complex multistage sampling design and to produce weighted estimates and 95% confidence intervals (CI).

For all comparisons we used a two-sided statistical test with significance defined as *p* value (*p*) <0.05 or a 95% CI that did not include the null value. The ggplot2 package of R was used to produce graphics [14].

## Results

From 1997 to 2006, the US population increased in total numbers and mean age, decreased in the proportion non-Hispanic white, and increased in prevalence of diabetes (all *p* values <0.001). The unweighted total number of deaths by year in the NHIS follow-up sample increased from 614 in year 2000 to 2,046 in 2006 (Table 2). The weighted total numbers of deaths from the NHIS follow-up (data not shown) were less than, but very close to, the total numbers of deaths of all US adults aged 20 to 84 years using the NVSS (Table 2).

**Table 2:** All-cause mortality rates of US adults aged 20 to 84 years (per 1,000 person-years and 95% CI) by calendar year using discretized survival time, NVSS and NHIS follow-up

	2000	2001	2002	2003	2004	2005	2006
National Vital Statistics System							
All-cause deaths, *n*	1,690,834	1,697,147	1,708,100	1,706,555	1,672,235	1,691,092	1,671,006
Population, *n*	196,709,054	199,749,920	202,082,985	204,215,941	206,505,061	208,818,040	211,189,565
All-cause mortality (M0)^a^	8.60	8.50	8.45	8.36	8.10	8.10	7.91
NHIS mortality follow-up with the post-stratification sampling weights							
All-cause deaths, *n*	614	874	1,124	1,417	1,638	1,869	2,046
Sample adults, *n*	60,395	86,722	114,050	141,678	166,593	190,342	214,530
Person-years	60,070	86,274	113,501	140,958	165,769	189,394	212,803
All-cause mortality (M1)^b^	8.81 (8.00, 9.62)	8.45 (7.75, 9.14)	8.35 (7.77, 8.93)	8.39 (7.89, 8.89)	7.98 (7.54, 8.42)	8.11 (7.65, 8.58)	7.94 (7.56, 8.33)
NHIS mortality follow-up with original sampling weights							
All-cause mortality (M2)^b^	8.99 (8.17, 9.82)	8.80 (8.08, 9.52)	8.67 (8.07, 9.28)	8.83 (8.31, 9.36)	8.41 (7.95, 8.87)	8.58 (8.10, 9.06)	8.36 (7.95, 8.77)
(M2 – M1)	0.18 (−0.97, 1.33)	0.35 (−0.65, 1.35)	0.32 (−0.53, 1.17)	0.44 (−0.29, 1.17)	0.43 (−0.19, 1.05)	0.47 (−0.18, 1.12)	0.42 (−0.15, 0.99)

^a^All-cause mortality from the NVSS was calculated as the number of the US all-cause deaths divided by the total US adults

^b^All-cause mortality from the NHIS was calculated as the weighted number of deaths divided by the total weighted person-years

To show the importance of post-stratification reweighting, we compared the NHIS follow-up estimates with the results from NVSS and the NHIS estimates that used the original weights (Table 2). Mortality estimates using the original sampling weights without post-stratification adjustment were higher than the mortality estimates using adjusted sampling weights, because of the aging of cohorts during the follow-up. Mortality in each year from the NVSS was within the 95% CIs of mortality rates from the NHIS using the adjusted sampling weights. The average annual decrease in crude mortality (per 1,000 person-years) was 0.12 for both the NHIS and the NVSS. The correlation of NHIS and the NVSS mortality was 0.94. Age-sex-race/ethnicity-adjusted mortality decreased 2.6% per year (*p* < 0.001).

The all-cause mortality rate among adults with diabetes was 1.8 per 1,000 person-years, which is much higher than the diabetic mortality rate of 0.3 per 1,000 person-years. Table 3 shows that annual diabetic mortality rates using NHIS (M1) (range: 0.25-0.42) were similar to mortality using NVSS (M0) (range: 0.27-0.29), while all-cause mortality rates among those with self-reported diabetes (M2) were much higher (1.66-1.94). Among adults with diabetes, age-sex-race/ethnicity adjusted mortality decreased 3.7% per year (*p* = 0.011). Diabetic mortality (M3) accounted for approximately 13.0% (95% CI: 12.8%, 13.2%) of the all-cause mortality (M4). Among adults without diabetes at baseline, 0.08 per 1,000 person-years died from diabetes (M5).

**Table 3:** Diabetic^a^ and all-cause with diabetes^b^ mortality rates (per 1,000 person-years and 95% CI) of US adults aged 20 to 84 years by calendar year using discretized survival time data, NVSS and NHIS follow-up

	2000	2001	2002	2003	2004	2005	2006
National Vital Statistics System							
Diabetic death, *n*	55,661	57,105	58,431	59,164	58,123	59,108	57,260
Population, *n*	196,709,054	199,749,920	202,082,985	204,215,941	206,505,061	208,818,040	211,189,565
Diabetic mortality (M0)^c^	0.28	0.29	0.29	0.29	0.28	0.28	0.27
NHIS mortality follow-up: total adults with and without diabetes							
Diabetic deaths, *n*	24	32	55	61	55	79	69
Diabetic mortality (M1)^d^	0.26 (0.14, 0.37)	0.29 (0.18, 0.41)	0.42 (0.28, 0.57)	0.34 (0.23, 0.44)	0.26 (0.18, 0.34)	0.30 (0.21, 0.38)	0.25 (0.18, 0.33)
All-cause with diabetes death, *n*	123	196	249	310	344	408	440
All-cause with diabetes mortality (M2)^e^	1.78 (1.43, 2.13)	1.94 (1.59, 2.30)	1.80 (1.51, 2.09)	1.80 (1.55, 2.05)	1.63 (1.42, 1.83)	1.70 (1.51, 1.88)	1.66 (1.48, 1.83)
NHIS mortality follow-up: adults with diagnosed diabetes							
Diabetic deaths, *n*	15	22	45	51	44	56	47
Diabetic mortality (M3)^f^	3.50 (1.48, 5.51)	4.04 (2.08, 6.01)	7.03 (4.34, 9.72)	5.76 (3.85, 7.67)	4.04 (2.57, 5.51)	4.41 (2.94, 5.88)	3.06 (2.09, 4.03)
All-cause deaths, *n*	123	196	249	310	344	408	440
All-cause mortality (M4)^g^	35.7 (28.4, 42.9)	39.5 (32.4, 46.5)	36.1 (30.4, 41.8)	35.6 (30.7, 40.5)	31.7 (27.8, 35.6)	33.0 (29.4, 36.5)	31.8 (28.5, 35.1)
M3/M4, %	9.80 (9.05, 10.56)	10.13 (9.39, 10.86)	19.39 (18.76, 20.02)	16.29 (15.76, 16.82)	12.62 (12.20, 13.03)	13.37 (13.00, 13.75)	9.75 (9.40, 10.10)
NHIS mortality follow-up: adults without diagnosed diabetes							
Diabetic death, *n*	9	10	10	10	11	23	22
Diabetic mortality (M5)^h^	0.09 (0.03, 0.15)	0.10 (0.03, 0.17)	0.08 (0.03, 0.13)	0.05 (0.02, 0.08)	0.05 (0.02, 0.09)	0.07 (0.04, 0.11)	0.10 (0.05, 0.16)

^a^Diabetic death was defined as a person had a underlying cause of death as diabetes (ICD-10: E10-E14)

^b^All-cause with diabetes death was defined as a person with diabetes and died of any cause

^c^Diabetic mortality from the NVSS was calculated as the total number of diabetic death divided by the total number of US adults

^d^Diabetic mortality from the NHIS mortality follow-up was calculated as the weighted number of diabetic death divided by the weighted person-years of adults with and without diabetes

^e^All-cause with diabetes mortality from the NHIS mortality follow-up was calculated as the weighted number of deaths with diabetes divided by the weighted person-years of total adults

^f^Among adults with diabetes, diabetic mortality from the NHIS mortality follow-up was calculated as the weighted number of diabetic death divided by the weighted person-years of adults with diabetes

^g^Among adults with diabetes, all-cause mortality from the NHIS mortality follow-up was calculated as the weighted number of all-cause deaths divided by the weighted person-years of adults with diabetes

^h^Among adults without diabetes, diabetic mortality from the NHIS mortality follow-up was calculated as the weighted number of diabetic death divided by the weighted person-years of adults without diabetes

**Fig. 2:**
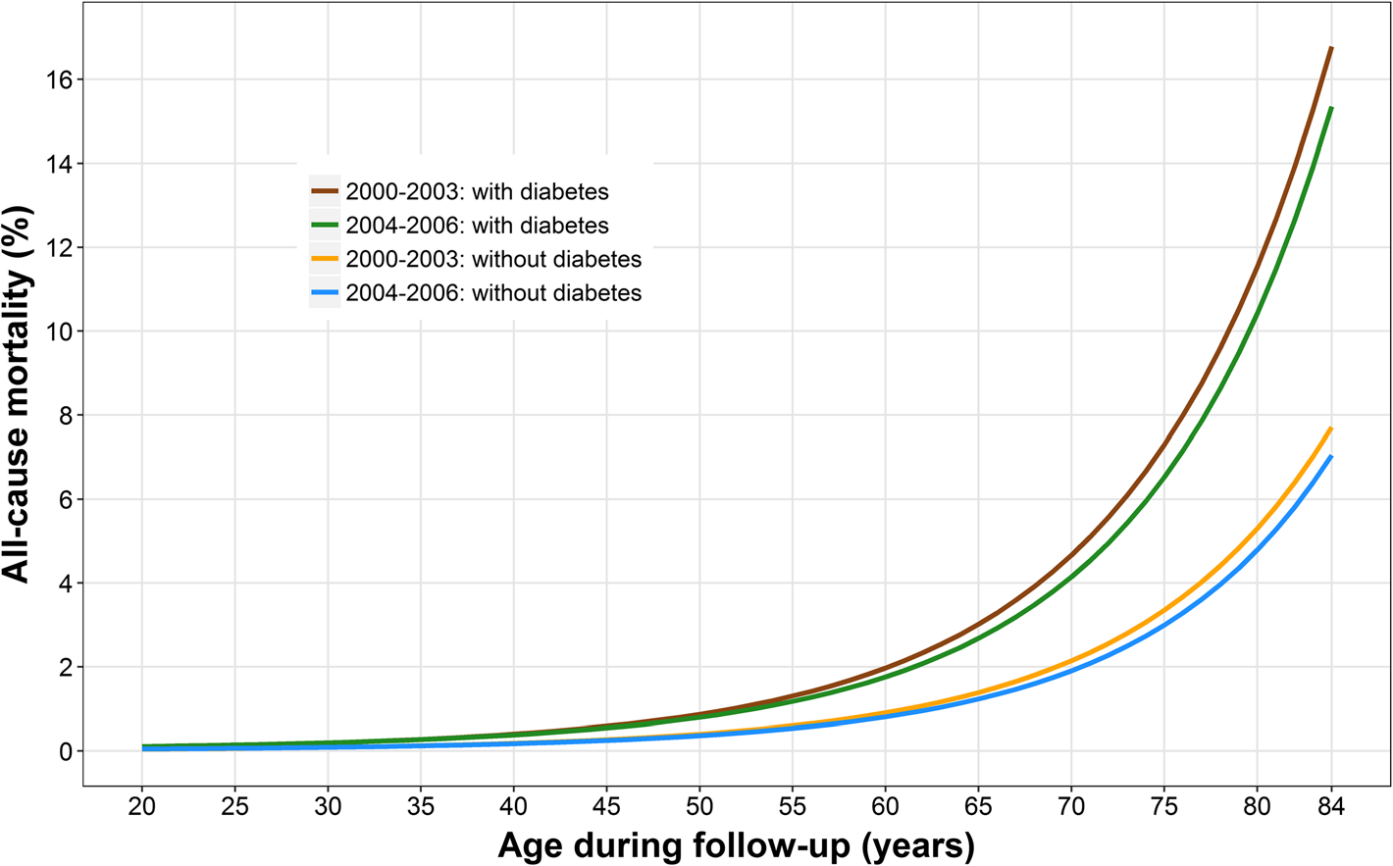
Sex-race/ethnicity-adjusted, age-specific, all-cause mortality by diabetes status estimated by polynomial Poisson regression

To demonstrate the flexibility of our approach, we calculated the sex-race/ethnicity-adjusted, age-specific, all-cause mortality by diabetes status at baseline using polynomial Poisson regression (Fig. 2). In summary, adults with diabetes at baseline had 2.31 (95% CI: 2.12, 2.50) times the risk of death compared with adults without diabetes (after adjusting for sex and race/ethnicity).

## Discussion

Period mortality among persons with chronic conditions such as diabetes is an important surveillance indicator of disease prevention and control. However, since chronic disease status is not reported in many vital statistics registries, it is often not possible to use vital statistics data to estimate mortality of persons with and without the condition. This presents a particular limitation for diabetes-related death statistics because diabetic death is often not recorded on US death certificates as a direct underlying or contributing cause of death, and diabetic deaths in the US population could be underestimated by solely using death certificate information [1, 2]. Assembly of national cohorts by linking national survey data with vital statistics provides a potential remedy to the data gap, but requires specific methods to permit estimation of period effects. In this study, we described the use of weighted discrete Poisson regression to estimate national mortality rates by diabetes status using a complex sample survey, and we validated this approach using mortality registry estimates. Our study showed that all-cause mortality of US adults estimated by the NHIS mortality follow-up decreased 2.6% per year from 2000 to 2006, which was similar to mortality rates estimated using the NVSS mortality registry data. Meanwhile all-cause mortality of US adults with diabetes decreased 3.7% per year during the same period [1, 2].

The method that is most often used to analyze mortality cohort data is the Cox proportional hazards regression model, which is useful for analyzing the data from the association or cause-effect relationship perspective. However, it is cumbersome to use this method to calculate hazard rates for a large number of combinations of predictors. Alternatively, parametric survival models can be more convenient for predicting, but cannot deal easily with time-varying covariates [15].

Age-period-cohort (APC) analysis provides a third option. If a vital statistics registry includes complete information on disease status, the APC method can be used to estimate the annual/period mortality among persons with and without diabetes [4, 6, 11, 16–18]. In the US, diabetes status is not recorded in the national vital statistics registry system. So we cannot apply this method directly. However, US nationally representative survey mortality follow-up data provide information on both diabetes status and death status. The APC model and life table framework can be applied to these data.

The APC analysis has been applied in demography, social science, and disease surveillance research using cross-sectional registration or survey data for a long time [19]. The data are usually cross-sectional and grouped for data analyses. One of the major purposes of these studies was to separate the age, period, or cohort effects using cross-sectional data [20]. In our study, we applied the concept and analytic framework of this widely used APC model. Compared to traditional APC models, our study had several differences. First, our study used longitudinal national complex survey mortality follow-up data. Second, the purpose was to estimate period mortality, which is a sum of the age and cohort effects. Finally, to account for the aging of the cohort during follow-up, we post-stratified the aggregated multiple segments from different survey cohorts using the US population structure at each period.

Both Poisson and logistic regression can be used for discretized time-to-event data analysis. Efron combined the logistic regression with discrete time-to-event survival time by 1-month intervals and obtained direct estimates of the hazard rates [21]. A polynomial or spline model can be used to smooth out the random variation/noise. This partial logistic regression gives good estimates when the discrete time interval is small. Nevertheless, a Poisson regression that accounts for person-time of follow-up gives more accurate hazard rate estimates for longer discrete time intervals than a logistic regression. Poisson regression has been used frequently to compare mortality rates among different categories of cohorts in epidemiological studies and is a convenient alternative to Cox proportional hazards regression especially when the proportional hazards assumptions are not met [5]. Early studies on the analysis of cohort survival data showed that Poisson regression is a straightforward and intuitive approach for directly estimating the hazard rates while incorporating time scale as a covariate in the model [16, 22]. We were interested in annual (or longer) time periods rather than monthly or daily periods and thus discrete Poisson regression was chosen for our analysis.

To obtain valid national estimates from a complex sample survey, it is critical to use proper statistical methods to account for the sample design and sampling weights. Our study shows that in later years, the distribution of age in the follow-up cohort shifted to the right; thus without post-stratification reweighting, the overall mortality rates combining all ages would have been overestimated. Using the US population as the standard population for post-stratification re-weighting yielded all-cause and diabetic mortality estimates that were similar to the national registry estimates. Our study demonstrated that discrete Poisson regression with post-stratification is a feasible approach for estimating annual mortality for the US population with and without diabetes.

The major limitation of our approach is the amount of time needed to discretize and analyze a large sample with long follow-up time. Poisson regression using complex sample data is computationally time-consuming with large discretized person-time datasets because data cannot be collapsed over covariates to account for the design-based analysis of complex sample data. Estimation based on a small number of events can create problems with model convergence. Without careful programming and reweighting, the results can be biased. In addition, the NHIS mortality data represented deaths among the civilian non-institutionalized population with person-year as the denominator, whereas mortality data from the NVSS represented deaths among the entire US population with the whole population at risk as the denominator. Thus, mortality rates from the two systems might have subtle differences.

To demonstrate our approach, we used self-reported diabetes. While any self-reported condition is subject to recall error, the self-report of diabetes is considered a valid measure of diagnosed diabetes [23]. Although it is recognized as being non-sensitive, it has been shown to be highly specific [24]. Another source of bias may arise from the lack of information about diabetes status between the baseline interview and death or censoring. Even though the rate of remission from diabetes to non-diabetes is likely small [25], the lack of information on incident cases would likely lead to an overestimation of diabetes duration. Furthermore, if incident cases have a higher mortality rate than non-cases and a lower mortality rate than prevalent cases, then lacking this information on incidence could lead to an overestimation of mortality rates for the populations both with and without diabetes. Future analyses with information with multiple follow-up visits could quantify the impact of this bias. We demonstrated that weighted discrete Poisson regression is an efficient applicable approach to estimate period mortality from the national mortality follow-up data. To our knowledge, there has been no similar report, though all the steps of this approach are well established. Several reasons could explain the scant usage of the discrete Poisson regression approach, including lack of data availability, lack of its inclusion as part of biostatistics educational curricula, the computing time required to analyze discrete time-to-event data, and the complex sampling design of national surveys, which further complicates using this approach. However, the increasing availability of more powerful statistical software and computing capabilities permits a revisitation of this method for the analysis of national survey mortality follow-up data.

## Conclusions

We conclude that combining national follow-up cohorts from multiple survey years and analyzing them using population weighted discrete Poisson regression can yield annual national mortality rates by disease status.

## Abbreviations

CDC: Centers for disease control & prevention
CDC-WONDER: Wide-ranging online data for epidemiologic research
NCHS: National center for health statistics
NHIS: National health interview survey
NVSS: National vital statistics system

## References

[1] US Centers for Disease Control and Prevention. 2014 US National Diabetes Statistics Report Atlanta (GA): Centers for Disease Control and Prevention; [updated May 15, 2015; cited 2015 February 19, 2016.]. Available from: http://www.cdc.gov/diabetes/data/statistics/2014StatisticsReport.html.

[2] Miniño AM, Murphy SL, Xu J, Kochanek KD (2011). Deaths: final data for 2008. Natl Vital Stat Rep.

[3] Frost WH (1995). The age selection of mortality from tuberculosis in successive decades. 1939.. Am J Epidemiol.

[4] Keiding N (1990). Statistical Inference in the Lexis Diagram. Philos T Roy Soc A.

[5] Laird N, Olivier D (1981). Covariance analysis of censored survival data using log-linear analysis techniques. J Am Stat Assoc.

[6] Hansen MB, Jensen ML, Carstensen B (2012). Causes of death among diabetic patients in Denmark. Diabetologia.

[7] Gail MH, Graubard B, Williamson DF, Flegal KM (2009). Comments on ‘Choice of time scale and its effect on significance of predictors in longitudinal studies’ by Michael J. Pencina, Martin G. Larson and Ralph B. D'Agostino, Statistics in Medicine 2007; 26:1343–1359. Stat Med.

[8] Korn EL, Graubard BI, Midthune D (1997). Time-to-event analysis of longitudinal follow-up of a survey: choice of the time-scale. Am J Epidemiol.

[9] Pencina MJ, Larson MG, D’Agostino RB (2007). Choice of time scale and its effect on significance of predictors in longitudinal studies. Stat Med.

[10] US Centers for Disease Control and Prevention. About the National Health Interview Survey Hyattsville (MD): US Centers for Disease Control and Prevention; 2016 [updated October 8, 2016; cited 2016 February 19, 2016.]. Available from: http://www.cdc.gov/nchs/nhis/about_nhis.htm.

[11] Frome EL (1983). The analysis of rates using Poisson regression models. Biometrics.

[12] Zou G (2004). A modified poisson regression approach to prospective studies with binary data. Am J Epidemiol.

[13] Carter D, Signorino C (2010). Back to the future: Modeling time dependence in binary data. Polit Anal.

[14] Wickham H (2009). ggplot2: elegant graphics for data analysis.

[15] Reid N (1994). A Conversation with Sir David Cox. Stat Sci.

[16] Carstensen B, Kristensen JK, Ottosen P, Borch-Johnsen K (2008). Steering Group of the National Diabetes R. The Danish National Diabetes Register: trends in incidence, prevalence and mortality. Diabetologia.

[17] O’Brien RM (2000). Age period cohort characteristic models. Soc Sci Res.

[18] Bell FC, Miller ML. Life tables for the United States social security area 1900–1200 2005 [cited 2016 11/07]. Available from: https://www.ssa.gov/oact/NOTES/as120/LifeTables_Body.html.

[19] Vandeschrick C (2001). The Lexis diagram, a misnomer. Demogr Res.

[20] Keyes KM, Utz RL, Robinson W, Li G (2010). What is a cohort effect? Comparison of three statistical methods for modeling cohort effects in obesity prevalence in the United States, 1971–2006. Soc Sci Med.

[21] Efron B (1988). Logistic Regression, Survival Analysis, and the Kaplan-Meier Curve. J Am Stat Assoc.

[22] Breslow NE, Lubin JH, Marek P, Langholz B (1983). Multiplicative models and cohort analysis. J Am Stat Assoc.

[23] Jackson JM, DeFor TA, Crain AL, Kerby TJ, Strayer LS, Lewis CE (2014). Validity of diabetes self-reports in the Women’s Health Initiative. Menopause.

[24] Centers for Disease Control Prevention. National diabetes statistics report: estimates of diabetes and its burden in the United States, 2014: HHS CDC; 2014 [cited 2016 11/08]. Available from: http://www.cdc.gov/diabetes/pubs/statsreport14/national-diabetes-report-web.pdf.

[25] Gregg EW, Chen H, Wagenknecht LE, Clark JM, Delahanty LM, Bantle J (2012). Association of an intensive lifestyle intervention with remission of type 2 diabetes. JAMA.

